# Molecular Responses to the Zika Virus in Mosquitoes

**DOI:** 10.3390/pathogens7020049

**Published:** 2018-05-03

**Authors:** Catalina Alfonso-Parra, Frank W. Avila

**Affiliations:** 1 Max Planck Tandem Group in Mosquito Reproductive Biology, University of Antioquia, Calle 67 #53-108, Medellín 050010, Colombia; calfonso@ces.edu.co; 2 Instituto Colombiano de Medicina Tropical, Carerra 43A # 52 sur-99, Sabaneta 055450, Colombia

**Keywords:** Flavivirus, ZIKV, *Aedes aegypti*, *Aedes albopictus*

## Abstract

The Zika virus (ZIKV), originally discovered in 1947, did not become a major concern until the virus swept across the Pacific and into the Americas in the last decade, bringing with it news of neurological complications and birth defects in ZIKV affected areas. This prompted researchers to dissect the molecular interactions between ZIKV and the mosquito vector in an attempt to better understand not only the changes that occur upon infection, but to also identify molecules that may potentially enhance or suppress a mosquito’s ability to become infected and/or transmit the virus. Here, we review what is currently known regarding ZIKV-mosquito molecular interactions, focusing on ZIKV infection of *Aedes aegypti* and *Aedes albopictus*, the primary species implicated in transmitting ZIKV during the recent outbreaks.

## 1. Introduction

The Zika virus (ZIKV) (Flaviviridae, Flavivirus) has received much attention in recent years, largely due to major outbreaks in French Polynesia in 2013 and later Brazil in 2014, which brought news of neurological complications and an increased incidence of congenital birth defects in babies born in affected areas. Although these epidemics brought Zika into the spotlight, the virus was originally discovered in Uganda in 1947 and isolated from *Aedes africanus* mosquitoes the following year [1,2]. Serological and entomological evidence suggests that ZIKV infections occurred on the African continent, Southeast Asia and India since the initial discovery of the virus [3,4,5,6,7,8,9,10,11]. However, the virus remained obscure, with few human cases reported until the outbreak on Yap Island, Micronesia in 2007 [12], where ZIKV was identified in serum samples from island residents [12,13]. In addition to a few sporadic cases of ZIKV infection that followed the Yap island outbreak [14,15], ZIKV spread throughout the Pacific [16] and caused a major outbreak in French Polynesia in late 2013–early 2014 [17,18].

The next major ZIKV outbreak, and the first in the Americas, occurred in Northeastern Brazil in late 2014 [19]. The virus spread quickly: by May 2015 at least 14 additional Brazilian states were affected [20]. The outbreaks in Brazil were particularly alarming due to the rise in cases of neurological disorders, such as Guillain–Barré syndrome, and the incidence of microcephaly in babies born in ZIKV-affected areas [21], leading WHO to declare a public health emergency of international concern in early 2016 [22]. A retrospective analysis suggests that microcephaly cases may also have been associated with the earlier outbreak in French Polynesia [23]. ZIKV continued to spread throughout the Caribbean, Central and South America [24] and arrived in the continental United States in 2016 [25].

Two lineages of ZIKV have been identified—Asian and African [26]—with the former responsible for the outbreaks that occurred in the Americas [27] and implicated in causing the congenital defects reported during the Brazilian outbreak [28]. Although ZIKV has been isolated from numerous *Aedes* species (as well as some non-*Aedes* species) [29,30], transmission of ZIKV strain(s) of the recent outbreaks is thought to primarily occur through bites of infected *Aedes aegypti* and *Aedes albopictus* mosquitoes [31], vectors with wide ranging global distributions [32,33]. Indeed, transmission of ZIKV to humans by *Ae. aegypti* was first confirmed in the 1950s [34]. However, recent studies have indicated that *Ae. albopictus* is competent for ZIKV transmission [30], with evidence to suggest that this vector has transmitted ZIKV to humans as well [35]. *Aedes aegypti* and *Ae. albopictus* are also the primary vectors for the transmission of the dengue virus (DENV), a flavirius related to ZIKV [36,37].

With the emergence of ZIKV, the alarming complications of ZIKV infection in humans, and its transmission by mosquito vectors with worldwide distributions, studies that examine ZIKV–mosquito interactions have begun. While our collective knowledge of ZIKV-mosquito interaction is still limited, researchers have acted quickly to identify the mosquito molecular responses to ZIKV infection. In this review, we highlight the progress made in this area, such as the identification of immune pathways that respond to the virus and the transcriptional response to ZIKV infection. We will focus on *Ae. aegypti* and *Ae. albopictus*, species where researchers have concentrated their efforts [30,38], and the minimal evidence that other widely distributed mosquito species, such as *Culex* species, are able to transmit ZIKV [30,39,40,41,42].

## 2. Vector Competence of *Ae. aegypti* and *Ae. albopictus*

Mosquito infection by an arbovirus occurs when a female consumes a blood-meal from an infected host. Upon ingestion, the virus first infects the midgut, where it replicates and spreads to other tissues, including the hemolymph, fat body and salivary glands. After further replication, the virus can be transmitted to other hosts when the female consumes a subsequent blood-meal [43]. The ability of ZIKV to infect the female mosquito, disseminate to other tissues and ultimately be transmitted to a new host is dependent on the genetic background of both the mosquito and the virus.

Studies that have assessed the genetic diversity of distinct *Ae. aegypti* populations have found ample genetic variation among both common laboratory and field-derived strains [44,45]. Genetic analysis of *Ae. albopictus*, however, has revealed low levels of genetic variation in this species, likely resulting from passive, human assisted dispersal [46,47]; genetic variation in *Ae. albopictus* is greatest between long-established versus recently introduced populations [47,48].

Examination of *Ae. aegypti* strains differing in their susceptibilities to DENV infection identified stark differences in basal levels of immunity-related transcripts [49], demonstrating genetic background influences mosquito resistance to arboviral infection. The ZIKV and *Aedes* strains used in the studies reviewed here are diverse and originate from numerous sources, which will affect a multitude of mosquito–virus molecular interactions. Vector competence studies of *Ae. aegypti* and *Ae. albopictus* further support the influence of genetic background on the ability of a mosquito to become infected by and potentially transmit ZIKV. Table 1 shows vector competence studies in *Ae. aegypti* and *Ae. albopictus* initiated after the Yap Island outbreak in 2007. The results of these studies demonstrate the differing susceptibility to ZIKV infection (and potential transmission) among *Ae. aegypti* and *Ae. albopictus* strains. The ZIKV strains used in these experiments also vary in their ability to infect these species (in the same strain), highlighting the complex interactions influenced by genetic background that occur between the virus and mosquito during infection.

## 3. Molecular Responses to ZIKV Infection

### 3.1. Antiviral Responses to ZIKV Infection

Mosquitoes rely on innate antiviral processes to limit viral propagation, including the production of reactive oxygen species (ROS), innate immune pathways, and RNA interference (RNAi) pathways. The consumption of a blood-meal by a female introduces a virus to tissues of the gut, where the virus initially replicates before dissemination to other tissues. Blood-meal digestion activates the antioxidant enzyme catalase in the midgut, hypothesized to minimize the oxidative stress that results from release of the pro-oxidant molecule heme from human blood during hemoglobin degradation [75]. This protection against oxidative stress facilitates DENV infection in *Ae. aegypti* [76]. However, no such affect is detected during ZIKV infection; silencing catalase, which alters redox homeostasis in the gut, has no effect on the outcome of ZIKV infection [76].

Some of the variation observed in the ability of ZIKV to infect female mosquitoes and subsequently be transmitted to a new host presumably results from an individual’s ability to combat the viral infection. The Toll and JAK-STAT pathways are key players in mosquito anti-viral defense [77] and are involved in the defense against DENV [78,79]. Anglero-Rodriguez et al. [58] found that activation of the Toll and JAK/STAT pathways significantly lowered the intensity of ZIKV infection compared to controls [58], suggesting that the Toll and JAK/STAT pathways defend against ZIKV in *Ae. aegypti*.

However, Etebari et al. [80] reported that induction of the JAK/STAT pathway did not affect ZIKV infection in *Ae. aegypti*, concluding that this pathway is not involved in ZIKV–mosquito interactions. Further, transgenic activation of the JAK/STAT pathway in the fatbody of *Ae. aegypti* by a blood-meal inducible promoter inhibited mosquito infection by several DENV serotypes [81], but did not influence the intensity of ZIKV infection. While it is apparent that innate immune pathways respond distinctly to different viruses, further analysis is required to clarify how the mosquito immune system, particularly the JAK/STAT pathway, responds to ZIKV infection.

Activation of the IMD pathway, which defends against Gram-negative bacteria [77], in the midgut by Sindbis virus (SINV) inhibits SINV replication there [82], showing that an immune pathway can indirectly affect viral proliferation upon ingestion of an infected blood-meal. However, Anglero-Rodriguez et al. [58] found that activation of the IMD pathway in *Ae. aegypti* did not significantly affect ZIKV infection.

ZIKV infection elicits an RNAi response in *Ae. aegypti* [83], a pathway that limits viral replication and dissemination in the mosquito [84]. Virus-derived small-RNAs (15–35 nt) were detected in whole bodies of mosquitoes orally infected with ZIKV by two days post-inoculation (dpi) and increased substantially by 7 dpi and 14 dpi. The majority of RNA reads at the later time points were 21 nt, the typical length of virus-derived short interfering RNAs (vsiRNAs) [83]. ZIKV was also found to induce production of vsiRNAs in *Ae. aegypti* cells at 48hrs post-infection [85]; ZIKV-specific 21 nt vsiRNAs that mapped across the genome were observed at this time. Further, in both studies, viral-derived PIWI RNAs (piRNAs; 25–30 nt) were detected [83,85], opening the possibility that viral-derived piRNAs also interact with the vector. However, the vpiRNA molecules observed in both ZIKV studies lacked the piRNA ping-pong signature [83,85], which is detected in mosquito cells during infection by other arboviruses (e.g., SINV and La Crosse virus) [86]. Thus, while it appears that RNAi pathways are important mosquito defenses against viral infection, these pathways require further investigation to elucidate how they interact with ZIKV.

### 3.2. Gene Expression Changes during ZIKV Infection

The introduction of infectious agents into the mosquito induces changes in gene expression (e.g., immunity genes that combat infection). Gene expression profiles in whole bodies of *Ae. aegypti* infected with ZIKV were characterized at 2, 7 and 14 dpi [80]. ZIKV infection resulted in greater than two-fold expression changes (in either direction) of 1332 genes at all time points examined [80]. The largest number of transcriptional changes were observed at seven days post inoculation while the biggest changes in gene expression (genes with 10-fold differential expression) were most numerous at two days post inoculation. Gene ontology analysis showed that most genes altered by ZIKV-infection were involved in metabolic and cellular processes, and in proteolysis. In comparison with *Ae. aegypti* infection by DENV, a previous study found that the largest number of genes altered was observed at four days post inoculation [87]. Further, five transcripts with large differential changes during ZIKV infection [80] were also identified within the top 20 differentially regulated transcripts during *Ae. aegypti* infection by DENV, yellow fever virus (YFV), and West Nile virus (WNV) [88,89].

Examining gene expression exclusively in the midgut at seven days post inoculation, ZIKV infection resulted in the up- and down-regulation of 148 and 75 genes, respectively [58]. The authors also examined the midgut response to DENV infection and found that, in comparison to ZIKV, 61% of the genes identified were uniquely responsive to ZIKV. Twenty-six genes that respond to both DENV and ZIKV in the midgut [58] are also regulated in the same direction during DENV, YFV, and WNV infection [89]. The midgut transcriptomic response to ZIKV-infection revealed a significant representation in immune-related genes, 13 of which were associated with mosquito immune pathways (i.e., Toll, IMD, JAK/STAT, and RNAi) [58]. Of these, ZIKV induced expression of dicer-2 and -6 genes putatively linked to the Toll pathway [58], offering further support for the RNAi and Toll pathways in limiting infection and dissemination of ZIKV. 

### 3.3. Changes in miRNA and lincRNA Expression during ZIKV Infection

Regulatory RNA profiles are altered during ZIKV infection in *Ae. aegypti*. microRNAs—small, non-coding RNAs involved in gene regulation—are known to play a role in virus–mosquito interaction [90]. Seventeen miRNAs were found to be differentially expressed in response to ZIKV infection across all time points examined (2, 7 and 14 dpi). The majority of the identified miRNAs were down-regulated during ZIKV infection [83]. A subset of the miRNAs identified also responded to DENV in *Ae. albopictus* [91], suggesting a partially overlapping response to flavivirus infection in *Aedes* species. Several immune genes (e.g., Toll-like receptor) are predicted targets of the differentially expressed miRNAs after ZIKV infection [83], supporting the idea that ZIKV modulates the mosquito immune response.

Long intergenic non-coding RNAs (lincRNAs)—molecules with diverse functions that include RNA stabilization and gene regulation [92]—also respond to ZIKV infection in *Ae. aegypti*. Four hundred eighty six lincRNAs were differentially expressed during ZIKV infection [80]. This included 80 lincRNAs that also responded to DENV infection in *Ae. aegypti* [93], suggesting common lincRNAs might be involved in the *Ae. aegypti* response to flavivirus infection. However, the role of the differentially expressed lincRNAs identified is not clear, with further investigation required to determine how lincRNAs interact with ZIKV during infection. 

### 3.4. Microbiome Changes during ZIKV Infection

The bacterial microbiome affects numerous physiological processes in *Ae*. *aegypti*, including larval development, nutritional status and reproductive processes [94,95,96]. Further, gut bacteria affect the ability of female mosquitoes to transmit viruses [97,98,99,100]. Thus, factors that modulate *Ae. aegypti* bacterial profiles would potentially influence vector competence of the organism. Villegas et al. [101] examined the bacterial microbiome of *Ae. aegypti* females fed sugar, blood or blood + ZIKV at three and seven days after an initial blood-meal. While fluctuations were observed in all experimental groups, a core microbiome (taxa that accounted for ~50% of identified bacteria) was detected across all groups. However, two non-core operational taxonomic units were identified only in the blood + ZIKV-fed females, suggesting that bacteria profiles are altered in ZIKV-infected mosquitoes. Further, and perhaps more importantly, the non-core bacteria identified—*Rhodobacteraceae* and *Desulfuromonadaceae—*may potentially be used as biomarkers for ZIKV infection [101].

### 3.5. Additional ZIKV–Mosquito Interactions

The rapid geographic expansion of ZIKV that occurred recently suggests that an adaptive change might have occurred in the virus. Liu et al. [102] found that infection prevalence in *Ae. aegypti* mosquitoes of the ZIKV strain associated with the outbreaks in the Americas (GZ01) was greater than a common strain from Southeast Asia (FSS13025). The higher infectivity of the GZ01 strain appears to result from an increase in secretion of the viral nonstructural protein 1 (NS1) in the human host due to a single base-pair mutation (A188V) in NS1 that occurred in the Asian lineage of the virus [102]. NS1 was previously shown to enhance flavivirus acquisition from an infected host and subsequently increased viral prevalence in *Ae. aegypti* mosquitoes [103]. The authors were able to increase NS1 secretion by making the A188V mutation in the ZIKV FSS13025 strain (FSS13025-A188V), which increased the infectivity of this strain in *Ae. aegypti* when compared to infection by the wild-type, non-mutant strain. This result echoes Tsetsarkin et al. [104], which showed a single amino acid substitution (A266V) in the chikungunya virus (CHIKV) envelope protein (E1) increased vector specificity of CHIKV; the A266V mutation in E1 increases membrane fusion of viral particles in *Ae. albopictus* [105]. Thus, the enhanced transmission efficiency of ZIKV from humans to *Ae. aegypti* and the resulting increase in infection prevalence of the mosquito offers one possible explanation for the re-emergence of ZIKV and its rapid spread throughout the Pacific and the Americas. 

While a viral protein secreted in the host increases the efficiency of mosquito infection when it takes a blood-meal from an infected host, a saliva protein in the mosquito enhances its ability to transmit ZIKV. Blood-feeding up-regulates the expression of the 15 kDa protein LTRIN in *Ae. aegypti* salivary glands. LTRIN was shown to facilitate the transmission of ZIKV by interfering with lymphotoxin signaling; LTRIN directly interacts with the lymphotoxin-β receptor (LTβR) and inhibits its activation [106], thereby affecting host defense as LTβR signaling plays a key role in defending against bacterial, virus and parasite infections [107]. Jin et al. [106] further showed that RNAi silencing of LTβR increased ZIKV load in cell culture, supporting the role of this pathway in the response to ZIKV. Thus, Liu et al. [102] and Jin et al. [106] highlight how viral and mosquito molecules can enhance ZIKV infection prevalence in mosquitoes and increase a mosquitoes’ ability to transmit the virus.

## 4. Conclusions

The neurological complications and birth defects associated with the recent ZIKV outbreaks added urgency to determine vector competence of distinct *Ae. aegypti* and *Ae. albopictus* strains and to elucidate mosquito molecular responses to ZIKV infection. Recent studies have started to examine mosquito immune pathways that respond to ZIKV, identified changes in gene expression during ZIKV infection, and have shown that bacterial profiles are altered by ZIKV infection. However, there is still much work to be done in determining how molecular pathways and specific molecules respond to ZIKV in the mosquito.

The genetic background of the mosquito and virus used in the studies reviewed here undoubtedly influenced the mosquito response to ZIKV infection observed, and should be taken into consideration when interpreting the results of these studies. Table 2 shows the *Ae. aegypti* strains assessed for their molecular response to ZIKV infection. These studies were conducted with common laboratory strains permissive to ZIKV infection. It would be interesting to see the response of field-derived strains and/or strains shown to be resistant to flavivirus infection, which would potentially give insight into the molecular pathways and/or other molecules that may impart resistance to ZIKV infection.

Finally, the molecular response to ZIKV infection has only been examined in *Ae. aegypti* to date. Future analyses will hopefully assess ZIKV–*Ae. albopictus* interactions due to the potential of this species to transmit ZIKV. The studies reviewed here are important first steps in understanding the molecular processes that occur upon consumption of ZIKV-infected blood and have started to dissect why certain *Aedes* strains are more susceptible to infection, adding to our overall understanding of the mosquito response to flavivirus infection.

## Figures and Tables

**Table 1:** Recent studies examining vector competence of *Aedes aegypti* (AE) and *Aedes albopictus* (AL). The *Aedes* and Zika Virus (ZIKV) strain used are listed. The tissues examined and the time points after ZIKV inoculation (days post inoculation (dpi) *) are shown. When possible, the mosquito generation used and the genBank accession number of the ZIKV strain are given.

Study	Species	Mosquito Strain (Generation Used)	ZIKV Strain (genBank Accession No.)	Vector Competence Parameters Assessed (Tissue(s); Time Points Examined)
Li et al. (2012) [50]	AE	Western Singapore (F1)	MR766 (AY632535)	Infection Rate; Potential Transmission Rate (midguts, salivary glands; 1–7, 10, 14 dpi)
Wong et al. (2013) [51]	AL	Western Singapore (F3)	MR766 (AY632535)	Infection Rate; Dissemination Rate; Potential Transmission Rate (midguts, salivary glands, saliva; 1–7, 10, 14 dpi)
Diagne et al. (2015) [52]	AE	Dakar, Senegal (F1) Kédougou, Senegal (F1)	ArD 128000 ArD 132912 ArD 157995 ArD 165522 HD 78788 MR766 (AY632535)	Infection Rate; Dissemination Rate; Potential Transmission Rate (bodies, legs/wings, saliva; 5, 10, 15 dpi)
Chouin-Carneiro et al. (2016) [53]	AE	Cayenne, French Guiana (F1) Baie-Mahault, Guadeloupe (F2) Pointe Chaudière, Martinique (F1) Orlando, USA (>F10) Rio de Janeiro, Brazil (F1)	NC-2014-5132	Infection Rate; Dissemination Rate; Potential Transmission Rate (abdomens, thoraxes, heads; 4 and 7 dpi) * * Rio de Janeiro strain at 4, 7, 14 dpi
	AL	Vero Beach, USA (F7) Rio de Janeiro, Brazil (F1)	NC-2014-5132	Infection Rate; Dissemination Rate; Potential Transmission Rate (abdomens, thoraxes, heads; 4 and 7 dpi) * * Vero Beach strain at 4, 7, 14 dpi
Di Luca et al. (2016) [54]	AL	Scalea, Italy	H/PF/2013	Infection Rate; Dissemination Rate; Potential Transmission Rate (bodies, legs/wings; 3, 4, 7, 11, 14, 18, 21 dpi and saliva; 4, 7, 11, 14, 18, 21 dpi)
	AE	Reynosa, Mexico	H/PF/2013	Infection Rate; Dissemination Rate; Potential Transmission Rate (bodies, legs/wings; 3, 4, 7, 11, 14, 18, 21 dpi and saliva; 4, 7, 11, 14, 18, 21 dpi)
Hall-Mendelin et al. (2016) [55]	AE	Townsville, Australia (F4)	MR766 (AY632535)	Infection Rate; Dissemination Rate; Potential Transmission Rate (bodies, legs/wings, saliva; 5, 7, 10, 14 dpi)
Weger-Lucarelli et al. (2016) [56]	AE	Poza Rica, Mexico (F11–F13)	PRVABC59 (KU501215) MR766 (AY632535) 41525 (KU955591)	Infection Rate; Dissemination Rate; Potential Transmission Rate (legs/wings, bodies, saliva; 7 and 14 dpi)
Richard et al. (2016) [57]	AE	Tahiti, French Polynesia (F16–F18)	PF13/251013-18 (KY766069)	Infection Rate; Dissemination Rate; Potential Transmission Rate (bodies, legs, saliva; 2, 6, 9, 14 and 21 dpi)
Angleró-Rodríguez et al. (2017) [58]		Rockefeller Orlando, USA	FSS13025 (KU955593)	Infection Rate; Dissemination Rate; Potential Transmission Rate (midguts, abdomens; 4, 7, 10, 14 dpi) and salivary glands (10, 14, 21 dpi)
Azar et al. (2017) [59]	AL	Rio Grande Valley, USA (F5)	FSS130125 (KU955593) MEX 1-7 (KX247632) DakAR 41525 (KU955591)	Infection Rate; Dissemination Rate; Potential Transmission Rate (legs, bodies, saliva; 3, 7, 14 dpi)
Azar et al. (2017) [59]	AL	Houston, USA (F2)	PB 81 (KU365780) MEX 1-7 (KX247632) PRVABC 59 (KX377337)	Infection Rate; Dissemination Rate; Potential Transmission Rate (legs, bodies, saliva; 3, 7, 21 dpi)
Azar et al. (2017) [59]	AL	Salvador, Brazil (F3)	PB 81 (KU365780) MEX 1-7 (KX247632)	Infection Rate; Dissemination Rate; Potential Transmission Rate (legs, bodies, saliva; 3, 7, 21 dpi)
Ciota et al. (2017) [60]	AL	Yaphank, USA (F5–F7)	HND 2016–19563 (KX906952) CAM strain FSS130325 (JN860885)	Infection Rate; Dissemination Rate; Potential Transmission Rate (legs, bodies, saliva; 14 and 21 dpi)
	AE	Poza Rica, Mexico (F7, F8)	HND 2016–19563 (KX906952) CAM strain FSS130325 (JN860885)	Infection Rate; Dissemination Rate; Potential Transmission Rate (legs, bodies, saliva; 14 and 21 dpi)
Costa-da-Silva et al. (2017) [61]	AE	Rockefeller Higgs white-eye RED	ZIKV^BR^	Infection Rate; Dissemination Rate; Detection Rate (bodies, heads, saliva; 7 and 14 dpi)
Duchemin et al. (2017) [62]	AE	Cairns, Australia (F6, F7, F11)	Cambodia 2010 (KU955593)	Infection Rate; Dissemination Rate; Potential Transmission Rate (midguts, heads, anterior thoraxes, carcasses, saliva; 14 dpi)
	AL	Hammond Island, Australia (F4, F9)	Cambodia 2010 (KU955593)	Infection Rate; Dissemination Rate; Potential Transmission Rate (midgut, heads, anterior thorax, carcass, saliva; 14 dpi)
Fernandes et al. (2017) [41]	AE	Rio de Janeiro (>F10)	ZIKVPE243	Infection Rate; Dissemination Rate; Potential Transmission Rate (bodies, heads, saliva; 7 and 14 dpi)
Fernandes et al. (2017) [41]	AE	Rio de Janeiro (>F10)	ZIKVSPH	Infection Rate; Dissemination Rate; Potential Transmission Rate (bodies, heads, saliva; 14 dpi)
Fernandes et al. (2017) [41]	AE	Rio de Janeiro (>F10)	ZIKVU1	Infection Rate; Dissemination Rate; Potential Transmission Rate (bodies, heads, saliva; 7 dpi)
Göertz et al. (2017) [63]	AE	Rockefeller	011V-01621 (KU937936)	Infection Rate; Potential Transmission Rate (bodies, saliva; 14 dpi)
Heitmann et al. (2017) [64]	AE	Heidelberg, Germany	ZIKV_FB-GWUH-2016 (KU870645)	Infection Rate; Potential Transmission Rate (bodies, saliva; 14 and 21 dpi)
	AL	Freiburg, Germany (F7) Calabria, Italy (F7)	ZIKV_FB-GWUH-2016 (KU870645)	Infection Rate; Potential Transmission Rate (bodies, saliva; 14 and 21 dpi)
Huang et al. (2017) [65]	AE	Higgs white-eye (>F20)	PRVABC59 (KX377337)	Infection Rate; Dissemination Rate (abdomens; secondary tissues; 7 and 14 dpi)
Li et al. (2017) [66]	AE	Haikou, China Ruili City, China	SZ01 (KU866423)	Infection Rate; Dissemination Rate; Potential Transmission Rate (midguts, salivary glands, ovaries; 2, 4, 6, 8, 10, 12, 16, 20, 24 dpi)
Liu et al. (2017) [67]	AL	Foshan, China Guangzhou, China	(KU820899.2)	Infection Rate; Dissemination Rate; Potential Transmission Rate (midguts, heads, salivary glands; 0, 4, 7, 14 dpi)
	AE	Haikou, China	(KU820899.2)	Infection Rate; Dissemination Rate; Potential Transmission Rate (midguts, heads, salivary glands; 0, 4, 7, 14 dpi)
Pompon et al. (2017) [68]	AE	Singapore	H/PF/2013 (KJ776791) BE H 815744	Infection Rate; Potential Transmission Rate (whole bodies, saliva; 7 and 14 dpi)
	AL	Singapore	H/PF/2013 (KJ776791) BE H 815744	Infection Rate; Potential Transmission Rate (whole bodies, saliva; 7 and 14 dpi)
Roundy et al. (2017) [69]	AE	Salvador, Brazil (F2) Rio Grande Valley, USA (F4) Dominican Republic (F6)	FSS 130125 (KU955593.1) DAK AR 41525 (KU955591.1) MEX 1–7 (KX247632.1)	Infection Rate; Dissemination Rate; Potential Transmission Rate (bodies, legs; 2 dpi and bodies, legs and saliva; 4, 7, 10, 14 dpi)
Ryckebusch et al. (2017) [70]	AE	French Polynesia	PF-25013-18	Infection Rate; Dissemination Rate; Potential Transmission Rate (midguts, salivary glands; 3, 5, 6, 8, 10, 14 dpi and saliva; 8, 10, 14, 17 dpi)
	AL	Nice, France	PF-25013-18	Infection Rate; Dissemination Rate; Potential Transmission Rate (midguts, salivary glands, saliva; 3, 6, 8, 10, 14 dpi and saliva; 8, 10, 14 dpi)
Vazeille et al. (2017) [71]	AL	Réunion Island (F1)	NC-2014-5132	Infection Rate; Dissemination Rate (bodies and heads; 3, 6, 9, 14 dpi)
Willard et al. (2017) [72]	AE	Chiapas, Mexico (F2, F3)	MR766 (LC002520) IbH 30656 (KU963574) SPH (KU321639.1) Mex 1-44 (KX856011.1)	Infection Rate; Dissemination Rate; Potential Transmission Rate (bodies, heads, saliva; 14–15 dpi)
Zhao et al. (2017) [73]	AE	Key West, USA (F9) Orlando, USA	PRVABC59 (KU501215.1)	Infection Rate; Dissemination Rate; Potential Transmission Rate (bodies, legs, saliva; 5, 7, 10 dpi)
Dodson et al. (2018) [74]	AE	Rockefeller	PRVABC59 (KU501215)	Infection Rate; Dissemination Rate; Potential Transmission Rate (legs, bodies, saliva; 7 dpi)
Uraki et al. (2018) [44]	AE	Orlando Ho Chi Minh Patilas	MEX2-81 (KX446950)	Infection Rate; Dissemination Rate (midguts and salivary glands; 7 and 10 dpi)

**Table 2:** Studies examining the molecular response to ZIKV in *Aedes aegypti* mosquitoes. The *Ae. aegypti* and ZIKV strain are given. When possible, the genBank accession number of the ZIKV strain is given.

Study	*Aedes aegypti* Strain	ZIKV Strain (genBank Accession No.)
Anglero-Rodriguez et al. (2017) [58]	Rockefeller	FSS13025 (KU955593)
Etebari et al. (2017) [80]	Galveston	MEX 1-7 (KX247632)
Jupatanakul et al. (2017) [81]	Rockefeller	FSS13025 (KU955593)
Oliveira et al. (2017) [76]	Red Eye	Unknown
Saldana et al. (2017) [83]	Galveston	MEX 1-7 (KX247632)
Jin et al. (2018) [106]	Unknown	SZ01 (KU866423)
Liu et al. (2018) [102]	Rockefeller	GZ01 (KU820898) FSS13025 (KU955593) FSS13025-A188V
Villegas et al. (2018) [101]	PP-Campos	SPH (KU321639)

## References

[1] Lessler J., Chaisson L.H., Kucirka L.M., Bi Q., Grantz K., Salje H., Carcelen A.C., Ott C.T., Sheffield J.S., Ferguson N.M. (2016). Assessing the global threat from Zika virus. Science.

[2] Dick G.W., Kitchen S.F., Haddow A.J. (1952). Zika virus. I. Isolations and serological specificity. Trans. R. Soc. Trop. Med. Hyg..

[3] Kokernot R.H., Casaca V.M., Weinbren M.P., McIntosh B.M. (1965). Survey for antibodies against arthropod-borne viruses in the sera of indigenous residents of Angola. Trans. R. Soc. Trop. Med. Hyg..

[4] Macnamara F.N. (1954). Zika virus: A report on three cases of human infection during an epidemic of jaundice in Nigeria. Trans. R. Soc. Trop. Med. Hyg..

[5] Dick G.W. (1953). Epidemiological notes on some viruses isolated in Uganda; Yellow fever, Rift Valley fever, Bwamba fever, West Nile, Mengo, Semliki forest, Bunyamwera, Ntaya, Uganda S and Zika viruses. Trans. R. Soc. Trop. Med. Hyg..

[6] Smithburn K.C. (1954). Neutralizing antibodies against arthropod-borne viruses in the sera of long-time residents of Malaya and Borneo. Am. J. Hyg..

[7] Smithburn K.C., Kerr J.A., Gatne P.B. (1954). Neutralizing antibodies against certain viruses in the sera of residents of India. J. Immunol..

[8] Fagbami A.H. (1979). Zika virus infections in Nigeria: Virological and seroepidemiological investigations in Oyo State. J. Hyg. (Lond.).

[9] McCrae A.W., Kirya B.G. (1982). Yellow fever and Zika virus epizootics and enzootics in Uganda. Trans. R. Soc. Trop. Med. Hyg..

[10] Marchette N.J., Garcia R., Rudnick A. (1969). Isolation of Zika virus from *Aedes aegypti* mosquitoes in Malaysia. Am. J. Trop. Med. Hyg..

[11] Olson J.G., Ksiazek T.G., Suhandiman, Triwibowo (1981). Zika virus, a cause of fever in Central Java, Indonesia. Trans. R. Soc. Trop. Med. Hyg..

[12] Duffy M.R., Chen T.H., Hancock W.T., Powers A.M., Kool J.L., Lanciotti R.S., Pretrick M., Marfel M., Holzbauer S., Dubray C. (2009). Zika virus outbreak on Yap Island, Federated States of Micronesia. N. Engl. J. Med..

[13] Lanciotti R.S., Kosoy O.L., Laven J.J., Velez J.O., Lambert A.J., Johnson A.J., Stanfield S.M., Duffy M.R. (2008). Genetic and serologic properties of Zika virus associated with an epidemic, Yap State, Micronesia, 2007. Emerg. Infect. Dis..

[14] Kwong J.C., Druce J.D., Leder K. (2013). Zika virus infection acquired during brief travel to Indonesia. Am. J. Trop. Med. Hyg..

[15] Buathong R., Hermann L., Thaisomboonsuk B., Rutvisuttinunt W., Klungthong C., Chinnawirotpisan P., Manasatienkij W., Nisalak A., Fernandez S., Yoon I.K. (2015). Detection of Zika Virus Infection in Thailand, 2012–2014. Am. J. Trop. Med. Hyg..

[16] Musso D., Nilles E.J., Cao-Lormeau V.M. (2014). Rapid spread of emerging Zika virus in the Pacific area. Clin. Microbiol. Infect..

[17] Hancock W.T., Marfel M., Bel M. (2014). Zika virus, French Polynesia, South Pacific, 2013. Emerg. Infect. Dis..

[18] Cao-Lormeau V.M., Roche C., Teissier A., Robin E., Berry A.L., Mallet H.P., Sall A.A., Musso D. (2014). Zika virus, French polynesia, South pacific, 2013. Emerg. Infect. Dis..

[19] Campos G.S., Bandeira A.C., Sardi S.I. (2015). Zika Virus Outbreak, Bahia, Brazil. Emerg. Infect. Dis..

[20] World Health Organization (2015). Zika virus outbreaks in the Americas. Wkly. Epidemiol. Rec..

[21] Schuler-Faccini L., Ribeiro E.M., Feitosa I.M.L., Horovitz D.D.G., Cavalcanti D.P., Pessoa A., Doriqui M.J.R., Neri J.I., Neto J.M.D., Wanderley H.Y.C. (2016). Possible Association between Zika Virus Infection and Microcephaly—Brazil, 2015. MMWR Morb. Mortal. Wkly. Rep..

[22] WHO 2016. http://www.who.int/mediacentre/news/statements/2016/emergency-committee-zika-microcephaly/en/.

[23] Cauchemez S., Besnard M., Bompard P., Dub T., Guillemette-Artur P., Eyrolle-Guignot D., Salje H., Van Kerkhove M.D., Abadie V., Garel C. (2016). Association between Zika virus and microcephaly in French Polynesia, 2013-15: A retrospective study. Lancet.

[24] Fauci A.S., Morens D.M. (2016). Zika Virus in the Americas—Yet Another Arbovirus Threat. N. Engl. J. Med..

[25] Likos A., Griffin I., Bingham A.M., Stanek D., Fischer M., White S., Hamilton J., Eisenstein L., Atrubin D., Mulay P. (2016). Local Mosquito-Borne Transmission of Zika Virus—Miami-Dade and Broward Counties, Florida, June-August 2016. MMWR Morb. Mortal. Wkly. Rep..

[26] Haddow A.D., Schuh A.J., Yasuda C.Y., Kasper M.R., Heang V., Huy R., Guzman H., Tesh R.B., Weaver S.C. (2012). Genetic characterization of Zika virus strains: Geographic expansion of the Asian lineage. PLoS Negl. Trop. Dis..

[27] Faria N.R., Azevedo R., Kraemer M.U.G., Souza R., Cunha M.S., Hill S.C., Theze J., Bonsall M.B., Bowden T.A., Rissanen I. (2016). Zika virus in the Americas: Early epidemiological and genetic findings. Science.

[28] Cugola F.R., Fernandes I.R., Russo F.B., Freitas B.C., Dias J.L., Guimaraes K.P., Benazzato C., Almeida N., Pignatari G.C., Romero S. (2016). The Brazilian Zika virus strain causes birth defects in experimental models. Nature.

[29] Musso D., Gubler D.J. (2016). Zika Virus. Clin. Microbiol. Rev..

[30] Epelboin Y., Talaga S., Epelboin L., Dusfour I. (2017). Zika virus: An updated review of competent or naturally infected mosquitoes. PLoS Negl. Trop. Dis..

[31] Calvez E., Guillaumot L., Millet L., Marie J., Bossin H., Rama V., Faamoe A., Kilama S., Teurlai M., Mathieu-Daude F. (2016). Genetic Diversity and Phylogeny of *Aedes aegypti*, the Main Arbovirus Vector in the Pacific. PLoS Negl. Trop. Dis..

[32] Kraemer M.U., Sinka M.E., Duda K.A., Mylne A.Q., Shearer F.M., Barker C.M., Moore C.G., Carvalho R.G., Coelho G.E., Van Bortel W. (2015). The global distribution of the arbovirus vectors *Aedes aegypti* and *Ae. albopictus*. eLife.

[33] Ding F., Fu J., Jiang D., Hao M., Lin G. (2018). Mapping the spatial distribution of *Aedes aegypti* and *Aedes albopictus*. Acta Trop..

[34] Bearcroft W.G. (1956). Zika virus infection experimentally induced in a human volunteer. Trans. R. Soc. Trop. Med. Hyg..

[35] Grard G., Caron M., Mombo I.M., Nkoghe D., Mboui Ondo S., Jiolle D., Fontenille D., Paupy C., Leroy E.M. (2014). Zika virus in Gabon (Central Africa)—2007: A new threat from *Aedes albopictus*?. PLoS Negl. Trop. Dis..

[36] Mulyatno K.C., Kotaki T., Yotopranoto S., Rohmah E.A., Churotin S., Sucipto T.H., Amarullah I.H., Wardhani P., Soegijanto S., Kameoka M. (2018). Detection and Serotyping of Dengue Viruses in *Aedes aegypti* and *Aedes albopictus* (*Diptera*: *Culicidae*) Collected in Surabaya, Indonesia from 2008 to 2015. Jpn. J. Infect. Dis..

[37] Effler P.V., Pang L., Kitsutani P., Vorndam V., Nakata M., Ayers T., Elm J., Tom T., Reiter P., Rigau-Perez J.G. (2005). Dengue fever, Hawaii, 2001–2002. Emerg. Infect. Dis..

[38] Vorou R. (2016). Zika virus, vectors, reservoirs, amplifying hosts, and their potential to spread worldwide: What we know and what we should investigate urgently. Int. J. Infect. Dis..

[39] Lourenco-de-Oliveira R., Marques J.T., Sreenu V.B., Atyame Nten C., Aguiar E., Varjak M., Kohl A., Failloux A.B. (2018). *Culex quinquefasciatus* mosquitoes do not support replication of Zika virus. J. Gen. Virol..

[40] Van den Hurk A.F., Hall-Mendelin S., Jansen C.C., Higgs S. (2017). Zika virus and *Culex quinquefasciatus* mosquitoes: A tenuous link. Lancet Infect. Dis.

[41] Fernandes R.S., Campos S.S., Ribeiro P.S., Raphael L.M., Bonaldo M.C., Lourenco-de-Oliveira R. (2017). *Culex quinquefasciatus* from areas with the highest incidence of microcephaly associated with Zika virus infections in the Northeast Region of Brazil are refractory to the virus. Mem. Inst. Oswaldo Cruz.

[42] Guedes D.R., Paiva M.H., Donato M.M., Barbosa P.P., Krokovsky L., Rocha S., Saraiva K., Crespo M.M., Rezende T.M., Wallau G.L. (2017). Zika virus replication in the mosquito *Culex quinquefasciatus* in Brazil. Emerg. Microbes Infect..

[43] Franz A.W., Kantor A.M., Passarelli A.L., Clem R.J. (2015). Tissue Barriers to Arbovirus Infection in Mosquitoes. Viruses.

[44] Uraki R., Hastings A.K., Gloria-Soria A., Powell J.R., Fikrig E. (2018). Altered vector competence in an experimental mosquito-mouse transmission model of Zika infection. PLoS Negl. Trop. Dis..

[45] Gloria-Soria A., Ayala D., Bheecarry A., Calderon-Arguedas O., Chadee D.D., Chiappero M., Coetzee M., Elahee K.B., Fernandez-Salas I., Kamal H.A. (2016). Global genetic diversity of *Aedes aegypti*. Mol. Ecol..

[46] Schmidt T.L., Rasic G., Zhang D., Zheng X., Xi Z., Hoffmann A.A. (2017). Genome-wide SNPs reveal the drivers of gene flow in an urban population of the Asian Tiger Mosquito, *Aedes albopictus*. PLoS Negl. Trop. Dis..

[47] Sherpa S., Rioux D., Pougnet-Lagarde C., Despres L. (2018). Genetic diversity and distribution differ between long-established and recently introduced populations in the invasive mosquito *Aedes albopictus*. Infect. Genet. Evol..

[48] Kotsakiozi P., Richardson J.B., Pichler V., Favia G., Martins A.J., Urbanelli S., Armbruster P.A., Caccone A. (2017). Population genomics of the Asian tiger mosquito, *Aedes albopictus*: Insights into the recent worldwide invasion. Ecol. Evol..

[49] Sim S., Jupatanakul N., Ramirez J.L., Kang S., Romero-Vivas C.M., Mohammed H., Dimopoulos G. (2013). Transcriptomic profiling of diverse *Aedes aegypti* strains reveals increased basal-level immune activation in dengue virus-refractory populations and identifies novel virus-vector molecular interactions. PLoS Negl. Trop. Dis..

[50] Li M.I., Wong P.S., Ng L.C., Tan C.H. (2012). Oral susceptibility of Singapore *Aedes* (*Stegomyia*) *aegypti* (Linnaeus) to Zika virus. PLoS Negl. Trop. Dis..

[51] Wong P.S., Li M.Z., Chong C.S., Ng L.C., Tan C.H. (2013). *Aedes* (*Stegomyia*) *albopictus* (Skuse): A potential vector of Zika virus in Singapore. PLoS Negl. Trop. Dis..

[52] Diagne C.T., Diallo D., Faye O., Ba Y., Faye O., Gaye A., Dia I., Faye O., Weaver S.C., Sall A.A. (2015). Potential of selected Senegalese *Aedes* spp. mosquitoes (*Diptera*: *Culicidae*) to transmit Zika virus. BMC Infect. Dis..

[53] Chouin-Carneiro T., Vega-Rua A., Vazeille M., Yebakima A., Girod R., Goindin D., Dupont-Rouzeyrol M., Lourenco-de-Oliveira R., Failloux A.B. (2016). Differential Susceptibilities of *Aedes aegypti* and *Aedes albopictus* from the Americas to Zika Virus. PLoS Negl. Trop. Dis..

[54] Di Luca M., Severini F., Toma L., Boccolini D., Romi R., Remoli M.E., Sabbatucci M., Rizzo C., Venturi G., Rezza G. (2016). Experimental studies of susceptibility of Italian *Aedes albopictus* to Zika virus. Euro Surveill..

[55] Hall-Mendelin S., Pyke A.T., Moore P.R., Mackay I.M., McMahon J.L., Ritchie S.A., Taylor C.T., Moore F.A.J., van den Hurk A.F. (2016). Assessment of Local Mosquito Species Incriminates *Aedes aegypti* as the Potential Vector of Zika Virus in Australia. PLoS Negl. Trop. Dis..

[56] Weger-Lucarelli J., Ruckert C., Chotiwan N., Nguyen C., Garcia Luna S.M., Fauver J.R., Foy B.D., Perera R., Black W.C., Kading R.C. (2016). Vector Competence of American Mosquitoes for Three Strains of Zika Virus. PLoS Negl. Trop. Dis..

[57] Richard V., Paoaafaite T., Cao-Lormeau V.M. (2016). Vector Competence of French Polynesian *Aedes aegypti* and *Aedes polynesiensis* for Zika Virus. PLoS Negl. Trop. Dis..

[58] Anglero-Rodriguez Y.I., MacLeod H.J., Kang S., Carlson J.S., Jupatanakul N., Dimopoulos G. (2017). *Aedes aegypti* Molecular Responses to Zika Virus: Modulation of Infection by the Toll and Jak/Stat Immune Pathways and Virus Host Factors. Front. Microbiol..

[59] Azar S.R., Roundy C.M., Rossi S.L., Huang J.H., Leal G., Yun R., Fernandez-Salas I., Vitek C.J., Paploski I.A.D., Stark P.M. (2017). Differential Vector Competency of *Aedes albopictus* Populations from the Americas for Zika Virus. Am. J. Trop. Med. Hyg..

[60] Ciota A.T., Bialosuknia S.M., Zink S.D., Brecher M., Ehrbar D.J., Morrissette M.N., Kramer L.D. (2017). Effects of Zika Virus Strain and *Aedes* Mosquito Species on Vector Competence. Emerg. Infect. Dis..

[61] Costa-da-Silva A.L., Ioshino R.S., Araujo H.R., Kojin B.B., Zanotto P.M., Oliveira D.B., Melo S.R., Durigon E.L., Capurro M.L. (2017). Laboratory strains of *Aedes aegypti* are competent to Brazilian Zika virus. PLoS ONE.

[62] Duchemin J.B., Mee P.T., Lynch S.E., Vedururu R., Trinidad L., Paradkar P. (2017). Zika vector transmission risk in temperate Australia: A vector competence study. Virol. J..

[63] Goertz G.P., Vogels C.B.F., Geertsema C., Koenraadt C.J.M., Pijlman G.P. (2017). Mosquito co-infection with Zika and chikungunya virus allows simultaneous transmission without affecting vector competence of *Aedes aegypti*. PLoS Negl. Trop. Dis..

[64] Heitmann A., Jansen S., Luhken R., Leggewie M., Badusche M., Pluskota B., Becker N., Vapalahti O., Schmidt-Chanasit J., Tannich E. (2017). Experimental transmission of Zika virus by mosquitoes from central Europe. Euro Surveill..

[65] Huang Y.S., Lyons A.C., Hsu W.W., Park S.L., Higgs S., Vanlandingham D.L. (2017). Differential outcomes of Zika virus infection in *Aedes aegypti* orally challenged with infectious blood meals and infectious protein meals. PLoS ONE.

[66] Li C.X., Guo X.X., Deng Y.Q., Xing D., Sun A.J., Liu Q.M., Wu Q., Dong Y.D., Zhang Y.M., Zhang H.D. (2017). Vector competence and transovarial transmission of two *Aedes aegypti* strains to Zika virus. Emerg. Microbes Infect..

[67] Liu Z.Z., Zhou T.F., Lai Z.T., Zhang Z.H., Jia Z.R., Zhou G.F., Williams T., Xu J.B., Gu J.B., Zhou X.H. (2017). Competence of *Aedes aegypti*, Ae. albopictus, and *Culex quinquefasciatus* Mosquitoes as Zika Virus Vectors, China. Emerg. Infect. Dis..

[68] Pompon J., Morales-Vargas R., Manuel M., Tan C.H., Vial T., Tan J.H., Sessions O.M., Vasconcelos P.D., Ng L.C., Misse D. (2017). A Zika virus from America is more efficiently transmitted than an Asian virus by *Aedes aegypti* mosquitoes from Asia. Sci. Rep..

[69] Roundy C.M., Azar S.R., Rossi S.L., Huang J.H., Leal G., Yun R., Fernandez-Salas I., Vitek C.J., Paploski I.A., Kitron U. (2017). Variation in *Aedes aegypti* Mosquito Competence for Zika Virus Transmission. Emerg. Infect. Dis..

[70] Ryckebusch F., Berthet M., Misse D., Choumet V. (2017). Infection of a French Population of *Aedes albopictus* and of *Aedes aegypti* (Paea Strain) with Zika Virus Reveals Low Transmission Rates to These Vectors’ Saliva. Int. J. Mol. Sci..

[71] Vazeille M., Dehecq J.S., Failloux A.B. (2017). Vectorial status of the Asian tiger mosquito *Aedes albopictus* of La Reunion Island for Zika virus. Med. Vet. Entomol..

[72] Willard K.A., Demakovsky L., Tesla B., Goodfellow F.T., Stice S.L., Murdock C.C., Brindley M.A. (2017). Zika Virus Exhibits Lineage-Specific Phenotypes in Cell Culture, in *Aedes aegypti* Mosquitoes, and in an Embryo Model. Viruses.

[73] Zhao L., Alto B.W., Smartt C.T., Shin D. (2018). Transcription Profiling for Defensins of *Aedes aegypti* (*Diptera*: *Culicidae*) During Development and in Response to Infection With Chikungunya and Zika Viruses. J. Med. Entomol..

[74] Dodson B.L., Pujhari S., Rasgon J.L. (2018). Vector competence of selected North American *Anopheles* and *Culex* mosquitoes for Zika virus. PeerJ.

[75] Oliveira J.H., Goncalves R.L., Lara F.A., Dias F.A., Gandara A.C., Menna-Barreto R.F., Edwards M.C., Laurindo F.R., Silva-Neto M.A., Sorgine M.H. (2011). Blood meal-derived heme decreases ROS levels in the midgut of *Aedes aegypti* and allows proliferation of intestinal microbiota. PLoS Pathog..

[76] Oliveira J.H.M., Talyuli O.A.C., Goncalves R.L.S., Paiva-Silva G.O., Sorgine M.H.F., Alvarenga P.H., Oliveira P.L. (2017). Catalase protects *Aedes aegypti* from oxidative stress and increases midgut infection prevalence of Dengue but not Zika. PLoS Negl. Trop. Dis..

[77] Wang Y.H., Chang M.M., Wang X.L., Zheng A.H., Zou Z. (2017). The immune strategies of mosquito *Aedes aegypti* against microbial infection. Dev. Comp. Immunol..

[78] Souza-Neto J.A., Sim S., Dimopoulos G. (2009). An evolutionary conserved function of the JAK-STAT pathway in anti-dengue defense. Proc. Natl. Acad. Sci. USA.

[79] Ramirez J.L., Dimopoulos G. (2010). The Toll immune signaling pathway control conserved anti-dengue defenses across diverse *Ae. aegypti* strains and against multiple dengue virus serotypes. Dev. Comp. Immunol..

[80] Etebari K., Hegde S., Saldana M.A., Widen S.G., Wood T.G., Asgari S., Hughes G.L. (2017). Global Transcriptome Analysis of *Aedes aegypti* Mosquitoes in Response to Zika Virus Infection. mSphere.

[81] Jupatanakul N., Sim S., Anglero-Rodriguez Y.I., Souza-Neto J., Das S., Poti K.E., Rossi S.L., Bergren N., Vasilakis N., Dimopoulos G. (2017). Engineered *Aedes aegypti* JAK/STAT Pathway-Mediated Immunity to Dengue Virus. PLoS Negl. Trop. Dis..

[82] Barletta A.B., Nascimento-Silva M.C., Talyuli O.A., Oliveira J.H., Pereira L.O., Oliveira P.L., Sorgine M.H. (2017). Microbiota activates IMD pathway and limits Sindbis infection in *Aedes aegypti*. Parasites Vectors.

[83] Saldana M.A., Etebari K., Hart C.E., Widen S.G., Wood T.G., Thangamani S., Asgari S., Hughes G.L. (2017). Zika virus alters the microRNA expression profile and elicits an RNAi response in *Aedes aegypti* mosquitoes. PLoS Negl. Trop. Dis..

[84] Blair C.D., Olson K.E. (2015). The role of RNA interference (RNAi) in arbovirus-vector interactions. Viruses.

[85] Varjak M., Donald C.L., Mottram T.J., Sreenu V.B., Merits A., Maringer K., Schnettler E., Kohl A. (2017). Characterization of the Zika virus induced small RNA response in *Aedes aegypti* cells. PLoS Negl. Trop. Dis..

[86] Vodovar N., Bronkhorst A.W., van Cleef K.W., Miesen P., Blanc H., van Rij R.P., Saleh M.C. (2012). Arbovirus-derived piRNAs exhibit a ping-pong signature in mosquito cells. PLoS ONE.

[87] Bonizzoni M., Dunn W.A., Campbell C.L., Olson K.E., Marinotti O., James A.A. (2012). Complex modulation of the *Aedes aegypti* transcriptome in response to dengue virus infection. PLoS ONE.

[88] Londono-Renteria B., Troupin A., Conway M.J., Vesely D., Ledizet M., Roundy C.M., Cloherty E., Jameson S., Vanlandingham D., Higgs S. (2015). Dengue Virus Infection of *Aedes aegypti* Requires a Putative Cysteine Rich Venom Protein. PLoS Pathog..

[89] Colpitts T.M., Cox J., Vanlandingham D.L., Feitosa F.M., Cheng G., Kurscheid S., Wang P., Krishnan M.N., Higgs S., Fikrig E. (2011). Alterations in the *Aedes aegypti* transcriptome during infection with West Nile, dengue and yellow fever viruses. PLoS Pathog..

[90] Asgari S. (2014). Role of microRNAs in arbovirus/vector interactions. Viruses.

[91] Liu Y.X., Zhou Y.H., Wu J.Y., Zheng P.M., Li Y.J., Zheng X.Y., Puthiyakunnon S., Tu Z.J., Chen X.G. (2015). The expression profile of *Aedes albopictus* miRNAs is altered by dengue virus serotype-2 infection. Cell Biosci..

[92] Ransohoff J.D., Wei Y., Khavari P.A. (2018). The functions and unique features of long intergenic non-coding RNA. Nat. Rev. Mol. Cell Biol..

[93] Etebari K., Asad S., Zhang G.M., Asgari S. (2016). Identification of *Aedes aegypti* Long Intergenic Non-coding RNAs and Their Association with Wolbachia and Dengue Virus Infection. PLoS Negl. Trop. Dis..

[94] Minard G., Mavingui P., Moro C.V. (2013). Diversity and function of bacterial microbiota in the mosquito holobiont. Parasites Vectors.

[95] Gaio Ade O., Gusmao D.S., Santos A.V., Berbert-Molina M.A., Pimenta P.F., Lemos F.J. (2011). Contribution of midgut bacteria to blood digestion and egg production in aedes aegypti (*Diptera*: *Culicidae*) (L.). Parasites Vectors.

[96] Coon K.L., Brown M.R., Strand M.R. (2016). Gut bacteria differentially affect egg production in the anautogenous mosquito *Aedes aegypti* and facultatively autogenous mosquito *Aedes atropalpus* (*Diptera*: *Culicidae*). Parasites Vectors.

[97] Jupatanakul N., Sim S., Dimopoulos G. (2014). The insect microbiome modulates vector competence for arboviruses. Viruses.

[98] Hegde S., Rasgon J.L., Hughes G.L. (2015). The microbiome modulates arbovirus transmission in mosquitoes. Curr. Opin. Virol..

[99] Saldana M.A., Hegde S., Hughes G.L. (2017). Microbial control of arthropod-borne disease. Mem. Inst. Oswaldo Cruz.

[100] Guegan M., Zouache K., Demichel C., Minard G., Van V.T., Potier P., Mavingui P., Moro C.V. (2018). The mosquito holobiont: Fresh insight into mosquito-microbiota interactions. Microbiome.

[101] Villegas L.E.M., Campolina T.B., Barnabe N.R., Orfano A.S., Chaves B.A., Norris D.E., Pimenta P.F.P., Secundino N.F.C. (2018). Zika virus infection modulates the bacterial diversity associated with *Aedes aegypti* as revealed by metagenomic analysis. PLoS ONE.

[102] Liu Y., Liu J., Du S., Shan C., Nie K., Zhang R., Li X.F., Zhang R., Wang T., Qin C.F. (2017). Evolutionary enhancement of Zika virus infectivity in *Aedes aegypti* mosquitoes. Nature.

[103] Liu J., Liu Y., Nie K., Du S., Qiu J., Pang X., Wang P., Cheng G. (2016). Flavivirus NS1 protein in infected host sera enhances viral acquisition by mosquitoes. Nat. Microbiol..

[104] Tsetsarkin K.A., Vanlandingham D.L., McGee C.E., Higgs S. (2007). A single mutation in chikungunya virus affects vector specificity and epidemic potential. PLoS Pathog..

[105] Hoornweg T.E., van Duijl-Richter M.K.S., Ayala Nunez N.V., Albulescu I.C., van Hemert M.J., Smit J.M. (2016). Dynamics of Chikungunya Virus Cell Entry Unraveled by Single-Virus Tracking in Living Cells. J. Virol..

[106] Jin L., Guo X., Shen C., Hao X., Sun P., Li P., Xu T., Hu C., Rose O., Zhou H. (2018). Salivary factor LTRIN from *Aedes aegypti* facilitates the transmission of Zika virus by interfering with the lymphotoxin-beta receptor. Nat. Immunol..

[107] Spahn T.W., Eugster H.P., Fontana A., Domschke W., Kucharzik T. (2005). Role of lymphotoxin in experimental models of infectious diseases: Potential benefits and risks of a therapeutic inhibition of the lymphotoxin-beta receptor pathway. Infect. Immun..

